# P-1209. Efficacy of β-lactam Enhancer Based Zidebactam-Cefepime Combination (WCK 5222) versus Meropenem in Adults with Complicated Urinary Tract Infection (cUTI) or Acute Pyelonephritis (AP) in a Global, Randomized, Double-blind, Phase 3 Trial

**DOI:** 10.1093/ofid/ofaf695.1402

**Published:** 2026-01-11

**Authors:** Pencho Genov, Boris Mladenov, Donatas Slaitas, Mario Gonzalez, Pratikshit Mahajan, Haihui Huang, Denise Sharp, Alena Jandourek, Lily Llorens, David Friedland, Piotr Iwanowski, Ranjeet Gutte, Mahesh Patel, Sachin Bhagwat

**Affiliations:** University Multiprofile Hospital for Active Treatment "Kanev", Ruse, Ruse, Bulgaria; University Multiprofile Hospital for Active Teatment and Emergency Medicine - Pirogov (UMHATEM N.I.PIROGOV), Sofia, Sofiya, Bulgaria; Republican Vilnius University hospital, Vilnius, Vilniaus Apskritis, Lithuania; Santos Research Center, Corp, Tampa, Florida; Supe Heart and Diabetes Hospital And Research Centre, Nashik, Maharashtra, India; Fudan University Huashan Hospital, Institute of Antibiotics, Shanghai, Shanghai, China; Wockhardt, Aurangabad, Maharashtra, India; Wockhardt, Aurangabad, Maharashtra, India; Wockhardt, Aurangabad, Maharashtra, India; Wockhardt, Aurangabad, Maharashtra, India; Wockhardt, Aurangabad, Maharashtra, India; Wockhardt, Aurangabad, Maharashtra, India; Wockhardt Limited, Mumbai, Maharashtra, India; Wockhardt Limited, Mumbai, Maharashtra, India

## Abstract

**Background:**

The combination of β-lactam enhancer zidebactam and cefepime (ZID-FEP) is in development for the treatment of diverse infections caused by a wide range of MDR/XDR Gram-negative bacteria including MBL-producers. In a registration-enabling Phase 3 study (ENHANCE), efficacy and safety of ZID-FEP was compared against meropenem in adults with cUTI or AP.

**Figure:**
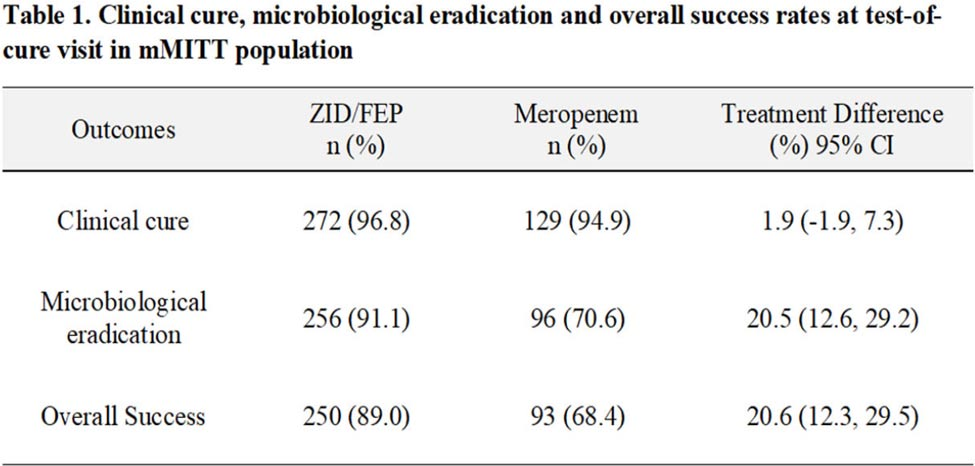
Clinical cure, microbiological eradication and overall success rates at test-of-cure visit in mMITT population

**Methods:**

This Phase 3, randomized, double-blind, active-controlled and non-inferiority trial was conducted at 44 global sites across U.S., EU, India, Mexico, and China, enrolling hospitalized adults (≥18 years old) with a clinical diagnosis of cUTI or AP ± concurrent bacteremia. Eligible patients were randomized 2:1 to receive ZID-FEP 1+ 2 g (1 h infusion) or meropenem 1 g (0.5 h infusion) for 7 to 10 days with dosage adjustments based on renal function. The primary efficacy outcome (FDA & EMA endpoint) was the proportion of patients in the Microbiological Modified Intent-To-Treat (mMITT) population who achieved overall success (clinical cure combined with microbiological eradication) at the test-of-cure (TOC) visit (Day 17±2). The non-inferiority margin was -15.0% and a pre-specified test for superiority for the primary endpoint was performed in the expanded-mMITT (e-mMITT) population following confirmation of non-inferiority (NCT04979806).

**Results:**

Of the 529 patients randomized and treated (352 in ZID-FEP and 177 in meropenem arms), 417 included in the mMITT population (281 in ZID-FEP and 136 in meropenem). Patients > 65 years represented 62% of the mMITT population and bacteremia was present in 6.5% of patients. The overall success rate at TOC was 89.0% in the ZID-FEP group versus 68.4% in the meropenem group; treatment difference [ZID-FEP versus meropenem], 20.6%; 95% CI, 12.3 to 29.5) (Table 1). ZID-FEP was statistically superior to meropenem for the primary endpoint at the TOC in e-mMITT. Treatment difference for all analyzed sub-groups, which include cUTI versus AP, older adults, renal-insufficiency, obese patients and cefepime-resistant uropathogens were consistent with the overall study results.

**Conclusion:**

ZID-FEP demonstrated statistical superiority over meropenem in the overall success rates and results were consistent regardless of patient or pathogen characteristics.

**Disclosures:**

All Authors: No reported disclosures

